# Management of gastroschisis in an extremely low birth weight infant: report of a case

**DOI:** 10.1186/s40792-024-02028-z

**Published:** 2024-10-09

**Authors:** Noboru Oyachi, Fuminori Numano, Tamao Shinohara, Yasushi Murakami, Atsushi Nemoto, Atsushi Naito

**Affiliations:** 1 Department of Pediatric Surgery, Yamanashi Prefectural Central Hospital, 1-1-1 Kofu, Yamanashi, 409-8506 Japan; 2 Department of Neonatology, Yamanashi Prefectural Central Hospital, Kofu, Japan

**Keywords:** Gastroschisis, Prematurity, Extremely low birth weight infant, Necrotizing enterocolitis, Bowel perfusion, Ileostomy, Silo placement

## Abstract

**Background:**

Gastroschisis is a rare congenital anomaly in which abdominal organs herniate through a defect in the abdominal wall. Managing gastroschisis in extremely low birth weight (ELBW) infants presents significant challenges because of their immature physiologies and increased risk of complications.

Case presentation: This report discusses the case of a female ELBW infant born via an emergency cesarean section at 29 weeks of gestation, weighing 768 g, who had a prenatal diagnosis of gastroschisis. Postnatal management included immediate surgical intervention using a hand-made silo manufactured from expanded polytetrafluoroethylene (ePTFE) sheets that were sutured to the patient’s abdominal wall to accommodate her small abdominal cavity and preserve mesenteric blood flow. Necrotizing enterocolitis with bowel perforation emerged as a complication, which led to the excision of a 10 cm segment of the ileum and the creation of an ileostomy. The infant experienced insufficient weight gain and liver dysfunction. However, she was eventually discharged on day 142 of life, weighing 2774 g, on oral feeding, without significant complications.

**Conclusions:**

This case emphasizes how prematurity significantly affected the patient’s clinical outcomes, and highlights the importance of individualized management strategies. Our experience demonstrates that custom silo placement allows for the size to be adapted to the abdominal defect, and highlights the critical need to prioritize postnatal bowel perfusion in ELBW infants with gastroschisis.

## Background

Gastroschisis is a rare congenital anomaly characterized by a distinct defect in the abdominal wall that develops early in embryonic development, typically located to the right of the umbilical cord [1]. In this condition, abdominal organs, predominantly the intestines, herniate through the defect. The condition is diagnosed prenatally and requires immediate surgical intervention after birth.

The incidence of gastroschisis has been increasing worldwide [2], with significant variations across different populations [3]. Managing gastroschisis in extremely low birth weight (ELBW) infants, defined as those weighing < 1000 g at birth, is particularly challenging, due to their immature physiologies and increased susceptibility to complications, such as delayed gastric emptying and a higher risk of sepsis, particularly from bowel perforation [4–6].

Herein, we present the case of a female ELBW infant delivered at 29 weeks of gestation, weighing 768 g. A detailed clinical presentation, including our surgical management approach, is accompanied by a review of the relevant literature.

## Case presentation

A 21-year-old primigravida woman with a history of smoking was referred to our institution at 17 weeks of gestation after a prenatal screening revealed fetal gastroschisis. The pregnancy was further complicated by intrauterine growth restriction, oligohydramnios, and non-reassuring fetal status, ultimately necessitating an emergency cesarean section at 29 weeks of gestation.

**Fig. 1:**
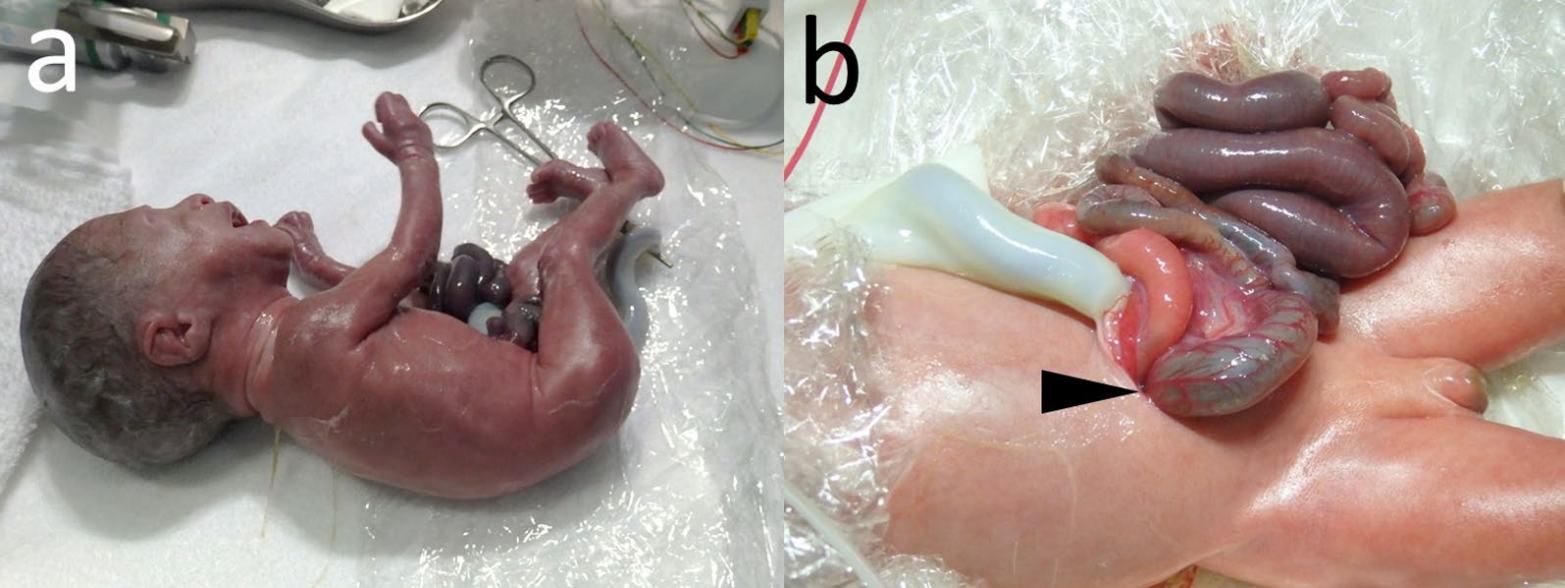
Abdominal wall defect at birth. **a** Female infant was born weighing 768 g, with Apgar scores of 5 and 8 at 1 and 5 min, respectively. **b** Abdominal wall defect measuring 10 mm in diameter was located to the right of the umbilical cord, with herniated small bowel and colon. The arrowhead indicates the defect

The female infant was born weighing 768 g, with Apgar scores of 5 and 8 at 1 and 5 min, respectively. She required immediate intubation and transfer to the neonatal intensive care unit. An abdominal wall defect measuring 10 mm in diameter was located to the right of the umbilical cord, with a herniated small bowel and colon (Fig. 1). Initially, the exposed bowel appeared viable; however, it showed signs of rapid swelling and ischemia shortly after birth.

**Fig. 2:**
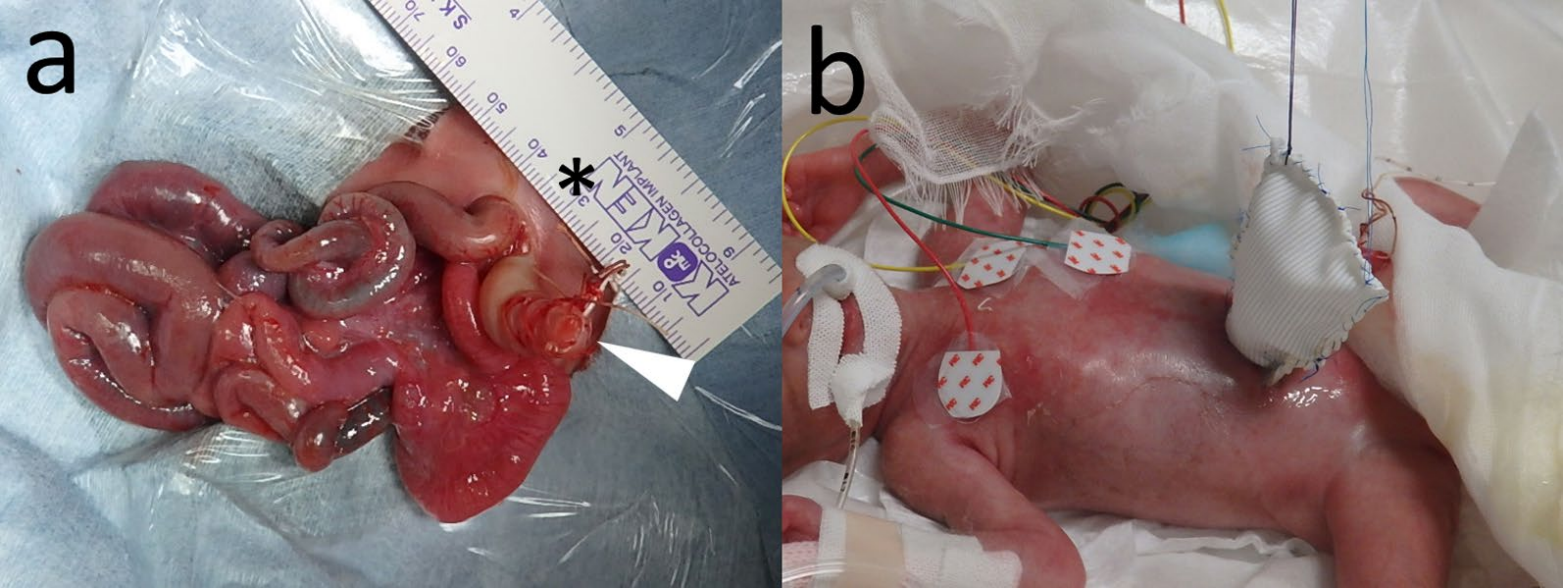
Emergency surgery and silo placement. **a** Emergency surgery was performed to relieve the pressure at the abdominal wall defect, which was constricting the pedicle of the herniated intestine. The arrowhead indicates the umbilicus, and the asterisk indicates the cranial direction. **b** Abdominal defect was enlarged through an upper midline incision extending 1 cm superiorly, and the “silo placement” technique was employed using expanded polytetrafluoroethylene (ePTFE) sheets, which were sutured to the abdominal wall

An emergency surgery was performed to relieve the pressure at the defect in the abdominal wall, which was constricting the pedicle of the herniated intestine. The abdominal defect was enlarged through an upper midline incision extending 1 cm superiorly, allowing for relaxation of the skin and fascia. No other vascular or intestinal complications were found (Fig. 2a). The "silo placement" technique was employed according to the Allen–Wrenn method [7], using expanded polytetrafluoroethylene (ePTFE) sheets that were sutured to the abdominal wall (Fig. 2b). The herniated intestines were gradually reduced into the peritoneal cavity via gentle compression and gravity.

**Fig. 3:**
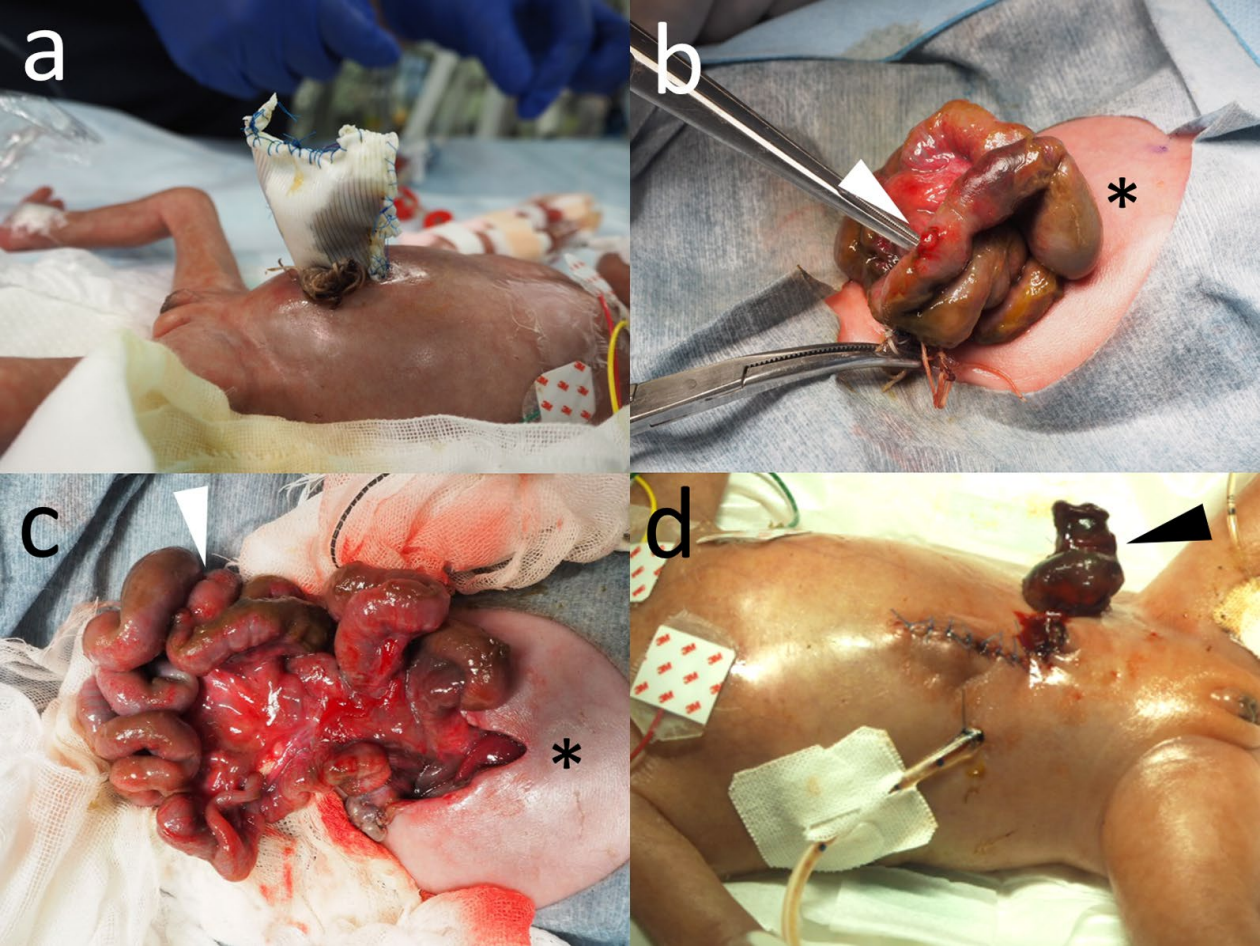
Bowel perforation with necrotizing enterocolitis. **a** On day 5 of life, bile-stained ascites leaked from the suture line between the silo and the abdominal wall, indicating bowel perforation. **b**, **c**, **d** Emergency laparotomy revealed necrotizing enterocolitis with localized bowel perforation in the exposed ileum in the silo, necessitating the excision of a 10 cm segment of the ileum and the creation of an ileostomy. The abdominal defect could be closed without increasing intra-abdominal pressure. The arrowhead indicates the umbilicus, and the asterisk indicates the cranial direction

Postoperative care initially progressed well, with half segments of the bowel successfully reduced. However, on day 5, bile-stained ascites leaked from the suture line between the silo and the abdominal wall, indicating bowel perforation (Fig. 3a).

An emergency laparotomy revealed necrotizing enterocolitis (NEC) with localized bowel perforation in the exposed ileum within the silo (Fig. 3b,c), necessitating the excision of a 10 cm segment of the ileum and the creation of an ileostomy. The abdominal defect could be closed without increasing intra-abdominal pressure (Fig. 3d). Pathological findings revealed transmural necrosis at the site of the perforation, accompanied by surrounding transmural inflammation and hemorrhage, consistent with NEC.

Postoperative peritonitis and septicemia developed, both of which were successfully treated with antibiotics. The infant required ventilation support by day 23 of life, transitioning from intubation to continuous positive airway pressure management. Nasogastric milk feeding was initiated on day 13 of life, but progressed slowly because of the patient’s gastrointestinal dysmotility. High-calorie parenteral nutrition was administered to support growth, but elevated serum bilirubin levels and transaminases, suggestive of liver dysfunction, were observed beginning on day 50 and peaking on day 97 (total bilirubin, 9.4 mg/dL; direct bilirubin, 6.7 mg/dL; AST, 120 IU/L; ALT, 69 IU/L). The patient's liver function subsequently improved and normalized by day 140, when enteral feeding was achieved. Ileostomy closure was performed on day 83 when the infant's weight exceeded 2000 g.

Although insufficient body weight gain was observed during the postoperative course, the infant was discharged on day 142 following birth, weighing 2774 g. At the time of discharge, she was on oral feeding and showed no signs of significant complications. By 18 months of age, the patient has shown acceptable growth and remains free of abdominal symptoms.

## Discussion

Managing gastroschisis in ELBW infants presents significant medical challenges, owing to their immature physiologies and increased risks of complications. In this case, the infant’s prematurity significantly influenced her clinical outcomes.

In relevant literature, it is rare to find reports of infants with gastroschisis born before 32 weeks of gestation. A study by the US Children's Hospitals Neonatal Consortium found that only 2.9% of infants with gastroschisis were born before 32 weeks of gestation [8]. The same study also found that infants with gastroschisis born before 32 weeks had a higher mortality rate (11%) compared to counterparts with gastroschisis born at higher gestational ages (< 2%) [8]. On the other hand, a recent study by Pugh et al. found that ELBW infants with gastroschisis had similar risks of mortality and specific intestinal complications, such as surgical NEC, compared to preterm infants without gastroschisis [6].

However, research specifically focusing on gastroschisis in ELBW infants is limited, with only a few case reports available in English-language literature. The details of these cases [4, 5] are shown in Table 1. Although the complications observed in this series are mostly associated with typical ELBW infants, the timing and technique of surgery, as well as postoperative care, were tailored to each particular case of gastroschisis in ELBW infants.

**Table 1:** ELBW infants with gastroschisis: characteristics and outcomes in English-language case reports

Author	Birth (GA)	Sex	BW	Initial intervention	Subsequent intervention	Gastroschisis-related complication	LOS	Dc weight	Misc
Johnson [4] (2011)	24wk	M	760 g	Silastic silo (0–3 days)	Abdominal closure (3 days)	TPN-associated cholestasis Delayed gastric emptying Gastroesophageal reflux	107d	NA	
Johnson [4] (2011)	24wk	F	747 g	Silastic silo (0–10 h)	Transparent film dressing (10 h—“few days”)	Compartment syndrome TPN-associated cholestasis Microperforation of bowel Gastroesophageal reflux	148d	3546 g	
Labuz [5] (2020)	26wk	M	580 g	Silastic silo (0–14 days)	Human cadaveric skin (14 days–8 m) Abdominal closure (8 m)	Chronic graft rejection (6 m)	4 m re Ad (6 m)	NA	Died (9 m)
Presented case	29wk	F	768 g	ePTFE silo (0–5 days)	Ileostomy and abdominal closure (5 days) Stoma closure (83 days)	Necrotizing enterocolitis TPN-associated cholestasis	142d	2774 g	

*ELBW* extremely low birth weight, *BW* birth weight, *d* days, *h* hours, *TPN* total parenteral nutrition, *LOS* length of stay, *Dc* discharge, *wk* weeks, *m* months, *re Ad* readmission, *Misc* miscellaneous. *NA* not available

Our experience highlights the critical need to prioritize postnatal bowel perfusion in ELBW infants with gastroschisis. It is crucial to ensure the construction of a secure silo to prevent hypothermia and abdominal fluid loss. Since interventions using silastic spring silos or wound retractor devices may not be feasible because of the small sizes of these infants, hand-made silo placement using silastic or ePTFE sheets is preferred. This allows the size of the silo to be adapted according to the abdominal defect. Rapid reduction to the abdominal cavity should be avoided to preserve mesenteric blood flow and reduce the risk of NEC.

Early recognition, accurate assessment, and appropriate management are crucial for overcoming the challenge of intestinal crises associated with gastroschisis. In this case, an ileal perforation caused by NEC led to the excision of a 10 cm segment of the ileum and the creation of an ileostomy, resulting in reduced bowel volume in the abdominal cavity. This may represent one of the reasons why the abdominal defect was closed in our case without increasing intra-abdominal pressure. Negash et al. reported the same phenomenon, noting that an ileostomy could assist in the primary closure of gastroschisis in certain patient groups [9].

## Conclusion

The rising incidence of gastroschisis and its link to prematurity suggest a potential increase in cases of extreme prematurity in the future. This case emphasizes how prematurity significantly affected the patient’s clinical outcomes, and highlights the importance of individualized management strategies. Our experience shows that hand-made silo placement allows for the size to be adapted to the patient’s abdominal defect, and highlights the critical need to prioritize postnatal bowel perfusion in ELBW infants with gastroschisis. Recent advances in perinatal care have improved survival rates; however, further optimizing patient outcomes require continued research and refinement of surgical techniques.

## Data Availability

The data set supporting the conclusion of this article is included within the article.

## Abbreviations

ELBW: Extremely low birth weight
ePTFE: Expanded polytetrafluoroethylene
NEC: Necrotizing enterocolitis
AST: Aspartate aminotransferase
ALT: Alanine aminotransferase
